# Polymeric micelles loading with ursolic acid enhancing anti-tumor effect on hepatocellular carcinoma

**DOI:** 10.7150/jca.30865

**Published:** 2019-10-06

**Authors:** Meiling Zhou, Youping Yi, Li Liu, Yan Lin, Jian Li, Jinghua Ruan, Zhirong Zhong

**Affiliations:** 1 Department of Pharmaceutical Sciences, School of Pharmacy, Southwest Medical University, Luzhou, Sichuan 646000, China.; 2 Department of Pharmacy, The Affiliated Hospital of Southwest Medical University, Luzhou, Sichuan 646000, China.; 3 The First Affiliated Hospital, Guiyang University of Chinese Medicine, Guiyang 550001, China.; 4 Key Laboratory of Medical Electrophysiology, Ministry of Education, Institute of Cardiovascular Research of Southwest Medical University, Luzhou 646000, China.

**Keywords:** Ursolic acid, Polymeric micelles, Antitumor effect, Hepatocellular carcinoma

## Abstract

Ursolic acid (UA) is widely found in many dietary plants, which has been proved to be effective in cancer therapy. But unfortunately its hydrophobic property limits its clinical application. Polymer micelles (PMs) are constructed from amphiphilic block copolymers that tend to self-assemble and form the unique core-shell structure consisting of a hydrophilic corona outside and a hydrophobic inner core. PMs could entrap the hydrophobic substance into its hydrophobic inner core for solubilizing these poorly water-soluble drugs and it is widely applied as a novel nano-sized drug delivery system. This study aimed to develop the drug delivery system of UA-loaded polymer micelles (UA-PMs) to overcome the disadvantages of UA in clinical application thus enhancing antitumor effect on hepatocellular carcinoma. UA-PMs was prepared and characterized for the physicochemical properties. It was investigated the cell-growth inhibition effect of UA-PMs against the human hepatocellular carcinoma cell line HepG2 and human normal liver cell line L-02. UA-PMs was evaluated about the *in vivo* toxicity and the antitumor activity. We took a diblock copolymer of methoxy poly (ethylene glycol)-poly(L-lactic acid) (mPEG-PLA) as carrier material to prepare UA-PMs by the thin-film dispersion method. MTT assay and wound-healing assay were investigated to assess the inhibition effect of UA-PMs against HepG2 cells on cell-growth and cell-migration. Further, we chose KM mice for the acute toxicity experiment and assessed the antitumor effect of UA-PMs on the H22 tumor xenograft. UA-PMs could markedly inhibit the proliferation and migration of HepG2 cells. *In vivo* study showed that UA-PMs could significantly inhibit the growth of H22 xenograft and prolong the survival time of tumor-bearing mice. It demonstrated that UA-PMs possess great potential in liver cancer therapy and may enlarge the application of UA in clinical therapy.

## Introduction

Hepatocellular carcinoma (HCC) is one of the most frequent deadly malignancies worldwide with high recurrence and high transfer rate. Unfortunately, some current therapeutic methods like resection, transplantation and ablation for HCC are often ineffective due to tumor metastasis and poor patient conditions 1-3. Some therapies are just suitable for only small numbers of patients. Chemotherapy plays the important role in the treatment of liver cancer but many anti-cancer drugs showed toxicity and side effects, and the bioavailability and curative effect are usually unsatisfactory. Currently, traditional Chinese medicines 3, as an important source for novel drug discovery with low toxicity and high biological activities, have been used clinically with great potential in the treatment of malignant tumors. Among them, ursolic acid (3β-hydroxy-urs-12-en-28-oic acid, UA) as one kind of pentacyclic triterpenoid is widely distributed in a variety of natural plants, such as loquat and glossy privet fruit. UA shows a variety of biological effects like anti-tumor 4, 5, 6 and anti-angiogenesis 7. It has been proved to be effective in anti- hepatocellular carcinoma 8. Moreover, it is low toxicity and became the hot spot in the study of anticancer drugs in recent years 6, 9. Therefore, we chose UA to develop the drug delivery system of UA-loaded polymer micelles (UA-PMs) to overcome the disadvantages of UA in clinical application. However, UA is critically limited in clinical application by its poor water solubility that leads to poor bioavailability 10, short retention time and non-specific distribution *in vivo* 11. Therefore, it is urgent to develop a novel drug delivery system for overcoming the shortcomings and improving the therapeutic effect of UA.

In recent years, various formulations of UA such as carrier-based and carrier-free nanoparticles 8, 12, 13, pH-sensitive prodrug 14, 15 and oral formulation 16 have been developed. As we known, polymer micelles (PMs) are constructed from amphiphilic block copolymers that tend to self-assemble and form the unique core-shell structure consisting of a hydrophobic inner core and a hydrophilic corona outside in an aqueous medium. It is widely applied as a novel nano-sized drug delivery system due to its numerous advantages 17-22. For example, the hydrophobic drugs can be entrapped into the hydrophobic inner core for solubilizing these poorly water-soluble drugs and protecting them against premature degradation, thereby prolonging the circulation time in blood. And the hydrophilic outer core forms a hydrophilic steric barrier that can protect polymeric micelles from the nonspecific uptake by the reticulo-endothelial system (RES) to prolong the systemic circulation time and make contributions to the stabilization of micelles* in vivo*. What's more, PMs possess the small and uniform particle size (10-100 nm) and can passively accumulate at tumor tissues as a result of an enhanced permeation and retention (EPR) effect, thus enhancing the antitumor activity and decreasing the side effects. In addition, as the carrier material of PMs, methoxy poly (ethylene glycol)-poly(L-lactic acid) (mPEG-PLA), has the advantages of good biodegradability and biocompatibility. PLA chains as hydrophobic segments are popularly utilized on account of its biodegradability 23, non-toxicity and biocompatibility, providing a sustained and controlled delivery. PEG is the most commonly used hydrophilic block due to its good biocompatibility and low toxicity. Meanwhile, PEG corona can entangle water molecules to form the hydrophilic shell accounting for enough steric stability between micelles and make the polymer micelles be unrecognizable for RES in the spleen and liver 24, 25. Moreover, PEG is a biologically inert compound with many excellences such as solubility in aqueous and organic media, low toxicity and good excretion kinetics, which is regularly used in both cosmetic and pharmaceutical preparations and is considered safe for use by the Food and Drug Administration 26. Small PEG molecules can be eliminated by kidney because of their hydrophilicity and size 26-28.

Furthermore, there are several kinds of methods to prepare polymer micelles such as dialysis method, self-assembled solvent evaporation method, thin film dispersion method and so forth. In the preparation of polymer micelles, the solubility of polymers as the primary factor should be taken into consideration 29. Although the dialysis method is simple, it is time-consuming. The encapsulation efficiency and drug loading are limited by the types of organic solvents. The preparation of micelles by thin-film dispersion method has the advantages of short time-consuming, simple preparation process, and small particle size of prepared micelles 30-32. So, the method of thin-film dispersion is of simplicity and is a good choice for preparing PMs.

Therefore, based on those findings mentioned above we hypothesize that mPEG-PLA as a drug carrier could be used to prepare UA-loaded polymeric micelles (UA-PMs) thus overcoming the limitations of free UA and improving the efficacy of UA in the treatment of liver cancers *in vitro* and* in vivo*.

In this study, we prepared UA-PMs by the thin-film dispersion method and investigated the cell-growth inhibition effect of UA-PMs against the human hepatocellular carcinoma cell line HepG2 and human normal liver cell line L-02 as shown in the Graphical abstract. We also assessed the *in vivo* toxicity of UA-PMs in normal Kunming mice and the antitumor effect of UA-PMs in H22 tumor-bearing mice.

## Materials and methods

### Materials

Both ursolic acid and standard ursolic acid were obtained from Nanjing Zelang pharmaceutical technology corporation (Nanjing, China). mPEG_2000_-PLA_2000_were from Jinan Daigang Biomaterial corporation (Jinan, China). Pyrene was purchased from Macklin (Shanghai, China).

Dulbecco's Modified Eagle's Medium (DMEM) with high glucose and Rosewell Park Memorial Institute (RPMI) 1640 medium were purchased from HyClone (Logan City, Utah, USA). Fetal bovine serum (FBS) was obtained from Sijiqing (Hangzhou, China). Paraformaldehyde was purchased from Jinshan Chemical Company (Chengdu, China). Trypsin, penicillin G, streptomycin and phosphate-buffered saline were obtained from Gibco Invitrogen (Carlsbad, CA, USA). 3-(4,5-dimethylthiazol-2-yl)-2,5-diphenyltetrazolium bromide (MTT) was from Amresco (St. Louis, MO, USA). All other reagents and chemicals were of analytical grade and purchased from Keyang Biotechnology Corporation (Luzhou, China).

### Cells and animals

Male Kunming mice with an average weight of 20 ± 2 g were purchased from the Animal Experiment Research Center of the Southwest Medical University, Luzhou, China. Mice were allowed to acclimate for a week before the experiment. All animals were bred with standard pellet diet in a standard animal-grade room with five animals in each cage on a 12-h light/12-h dark cycle. The temperature was set as room temperature. All the animal experimental procedures were conducted according to with the guidelines and protocols (Permit No. 20160142) approved by the Committee on the Use and Care of Animals of Southwest Medical University.

Human hepatocellular carcinoma cell line HepG2, human normal liver cell line L-02 and mouse hepatocellular carcinoma cells line (H22) were got from Shanghai Cell Institute, China Academy of Sciences and preserved in our laboratory. HepG2 cells were cultured in DMEM supplemented with 10% FBS, both H22 cells and L-02 cells in RPMI 1640 with 10% FBS, 100 U/mL penicillin and 100 µg/mL streptomycin at 37 °C under an atmosphere containing 5% CO_2_ and saturated humidity.

### Preparation of UA-PMs

UA-PMs were prepared by the thin-film dispersion method. Briefly, both UA (4 mg) and mPEG_2000_-PLA_2000_ (40 mg) with a mass ratio of 1:10 were dissolved by acetonitrile (4 mL) in a round-bottomed flask. Then the acetonitrile was completely removed through rotary evaporation in a 60 °C water bath to obtain a thin polymer film containing UA. The resultant thin film was hydrated with 2 mL of NaCl solution (0.9%, pH 7.4) to get a crude dispersion of the micelles. The micellar solution was mixed by water-bath ultrasound for 15 min followed by stirring for one hour at room temperature and filtered through a 0.22 µm film to remove the un-encapsulated UA. The blank polymeric micelles were prepared by the same way except addition of UA. The resulting micellar solution was lyophilized and stored at 4 °C for later use 33-37.

### Physicochemical characterization of UA-PMs

### Size and zeta potential of UA-PMs

The size distribution, polydispersity index and zeta potential of micelles were determined by photon correlation spectroscopy (PCS) (NanoBrook 90Plus Zeta, Brookhaven Instruments Ltd., USA). Before measurement, the samples were diluted at a concentration of 1 mg/mL. The measurements were performed at 25 °C, at a fixed angle of 90. The zeta potential was derived from mobility of particles in electric field by applying the Smoluchowsky relationship. The measurement was set for 2 min in each turn (n = 3).

### Morphology detection by SEM

The morphology of micelles was detected by the scanning electron microscopy (SEM) detection. Before SEM measurement, the samples were diluted at a concentration of 1.2 mg/mL. Sample was put on stubs with carbon adhesive disks and silver DAG, sputter coated, and viewed in a S-4800F (HITACHI) scanning electron microscope under the parameter setting of HV 5.0 kv, mag 20.0k ×, WD 8.6 mm and mode SE, respectively.

### Quantitative determination of UA-PMs by HPLC

A method based on high-performance liquid chromatography (HPLC) was developed for determining the content of ursolic acid 38. Chromatographic separation was performed on an Agilent Zorbax SB-C18 column (150 mm × 4.6 mm, 5 mm). The flow phase was a mixture of acetonitrile and water (93:7, v/v) at a flow rate of 1.0 mL**/**min. It was monitored at 210 nm, and the injection volume was 10 μL. Samples were diluted with methanol and filtered through a 0.22-μm membrane before injection. The method was fully proved in terms of selectivity, linearity, precision, recovery and so on.

### Encapsulation efficiency and drug loading rate detections of UA-PMs

In order to determine the encapsulation efficiency (EE, %) and drug loading rate (DLR, %) of UA-PMs, the micellar solution loaded with UA was dissolved in methanol and analyzed by HPLC method. EE and DLR of UA-PMs were obtained according to the following formula as: *EE (%) = (W_total_-W_free_) /W_total_ × 100%; DLR (%) = (W_total_-W_free_) / W_UA-PMs_ × 100%.* Among them, W_total_ is the total amount of UA in UA-PMs formulation, W_free_ is the amount of free / un-encapsulated UA in the same amount of UA-PMs formulation.

### *In vitro* release profile of UA-PMs

The *in vitro* release profile of UA-PMs was evaluated by a dialysis method. In detail, at least three batches of newly prepared UA-PMs were separately placed into dialysis bags (MWCO = 3500) and submerged in 100 mL PBS medium (pH 7.4 and pH 5.5) with gentle continuous-stirring at 37 °C. release medium. At predesigned time points of 0.5, 1, 2, 4, 6, 8, 12, 14, 24, 48, 72, 96 h, 2 mL of the solution was taken out from the release medium and supplemented with fresh PBS. After filtration through a 0.22-μm membrane (Millipore), all samples were measured using HPLC method mentioned above.

### Detection of the *in vitro* activity of UA-PMs

### Cell-growth inhibition by MTT assay

The cell proliferation inhibitory effect of UA-PMs was performed against human hepatocellular carcinoma cell line HepG2 and human normal liver cell line L-02 by MTT assay. All cells were seeded into 96-well cell plates with 1 × 10^4^ cells/well and incubated for 24 h until reaching confluence. Then, they were treated with free UA and UA-PMs at different concentration of 2.5, 5, 10, 20, 30, 40, 50, 60, 80, 100 μmol/L using DMEM medium as negative control and 5-FU as positive control. After incubation for 24 h, 20 μL of MTT solution (5 mg/mL) was added and incubated for another 4 h in the dark. The supernatant medium was removed and the left MTT-formazan crystals were dissolved in 150 mL DMSO. Then the plate was incubated for 10 min at 37 °C with gentle shaking before determining the absorbance at 490 nm using Varioskan Flash instrument (Thermo Fisher, Waltham, MA, USA). The inhibitory rate of cell proliferation was obtained through the following formula as: *Inhibition Rate (%) = (1 - A_sample_ / A_control_) × 100%.* Each assay was conducted in triplicate.

### Cell-migration inhibition detection of UA-PMs by wound healing assay

Cellular migration is an essential process in cancer development. For the wound healing migration assay, three horizontal lines were evenly drawn with a mark pen by a ruler on the outside bottom of the 6-well plates for recording images of the wounds at the same location.

When the HepG2 cells came to the logarithmic growth phase, they were inoculated into the marked plates at the concentration of 5 × 10^5^ cells per well and cultured to 90% fusion state. A 10-μL micropipette tip was used to scratch the plate with three parallel vertical lines and the exfoliated cells were mildly washed away by PBS for three times. Then, the cells were pre-incubated with DMEM containing 2% FBS and then treated with various concentrations of UA and UA-PMs (20, 40, and 80 µmol/L) with 5-fluorouracil (5-FU) as a positive control and DMEM containing 2% FBS as the negative control.

At the different time points of 0, 24, and 48 h after scratch, wound images were captured using an inverted phase-contrast microscope for the measurement of wound width and three images per well were recorded. The migration ability of the HepG2 cells treated with drugs was determined by the ratio of the scratch width (SW) at 24, 48 h to the wound width at 0 h and the wound healing rate was estimated by the following equation: *Wound healing rates (%) = (SW_0 h_ - SW_24 h or 48 h_) / SW_0 h_* × *100%.*

### Investigation about the *in vivo* antitumor efficacy of UA-PMs

Murine H22 cells were inoculated in the Kunming mice for the *in vivo* study 7, 39-41. Briefly, H22 cells were sub-cultured and diluted to the concentration of 1.0 × 10^8^ cells/mL with 0.9% saline. Under sterile conditions, 0.5 mL of cells suspension was injected into the peritoneal cavities of mice. After one week, ascites was extracted from the tumor-bearing mice for collecting the H22 cells by centrifugation and the cells were suspended by saline. Then, 0.2 mL of the cell suspension (1.0× 10^7^ cells/mL) was inoculated subcutaneously into the right flank of each mouse. Five days post tumor transplantation, when the tumors grew to about 100 mm^3^, the H22 tumor-bearing mice were divided randomly into seven groups (n=10). The treatments were separately initiated by intraperitoneal injection every two days for six times with normal saline as control, blank PMs, UA (50 mg/kg), 5-FU (25 mg/kg), UA-PMs (25 mg/kg), UA-PMs (50 mg/kg), UA-PMs (100 mg/kg), in which the formulation of free UA was prepared by dissolving UA into PEG_400_ (0.2%) and further being diluted with saline before injection. At the same time, every treatment group was randomly divided into part A and part B (n=5). In part A, the mice were treated every two days for 6 times and were sacrificed by cervical dislocation on day 17. The tumors were weighed and subjected to pathological analysis. In part B, the mice were also given the same treatment but they were kept feeding to record the death dates of mice for survival rate analysis. All the mice were monitored every day and the two axes of tumor (L, longest axis; W, shortest axis) were measured using a vernier micrometer for measurement of tumor size after treatment. Tumor volume (mm^3^) was calculated as ½ (L x W^2^). Meanwhile, Antitumor activity was assessed by tumor growth inhibition (TGI), which is the mean tumor weight (MTW) of the treated group (TG) relative to the saline-treated control group (CG) on day 17. It was obtained according to the formula as: *TGI (%) =* (*MTW_CG_* -*MTW_TG_*) / *MTW_CG_*✕ 100 %. Further, to assess tumor necrosis areas, the tumor tissues were fixed in 4% paraformaldehyde and then embedded by paraffin. Paraffin sections were stained with hematoxylin and eosin (H&E) and analyzed by ImageJ software. The average necrosis rate was estimated by the following equation: *Average necrosis rate (%) = Tumor necrosis areas / Total tumor areas × 100%.*

### Statistical analysis

All experiments were performed at least three times with a minimum of three independent experiments. All values were expressed as mean ± standard deviation (SD). One-way analysis of variance (ANOVA) or Student't-test was performed for statistical analysis between different groups. The Kaplan-Meier method was used in survival analysis. *P <* 0.05 was considered to be statistically significant, and *P <* 0.01 was considered as highly significant in all cases.

## Results

### Size and zeta potential of UA-PMs

**Figure 1:**
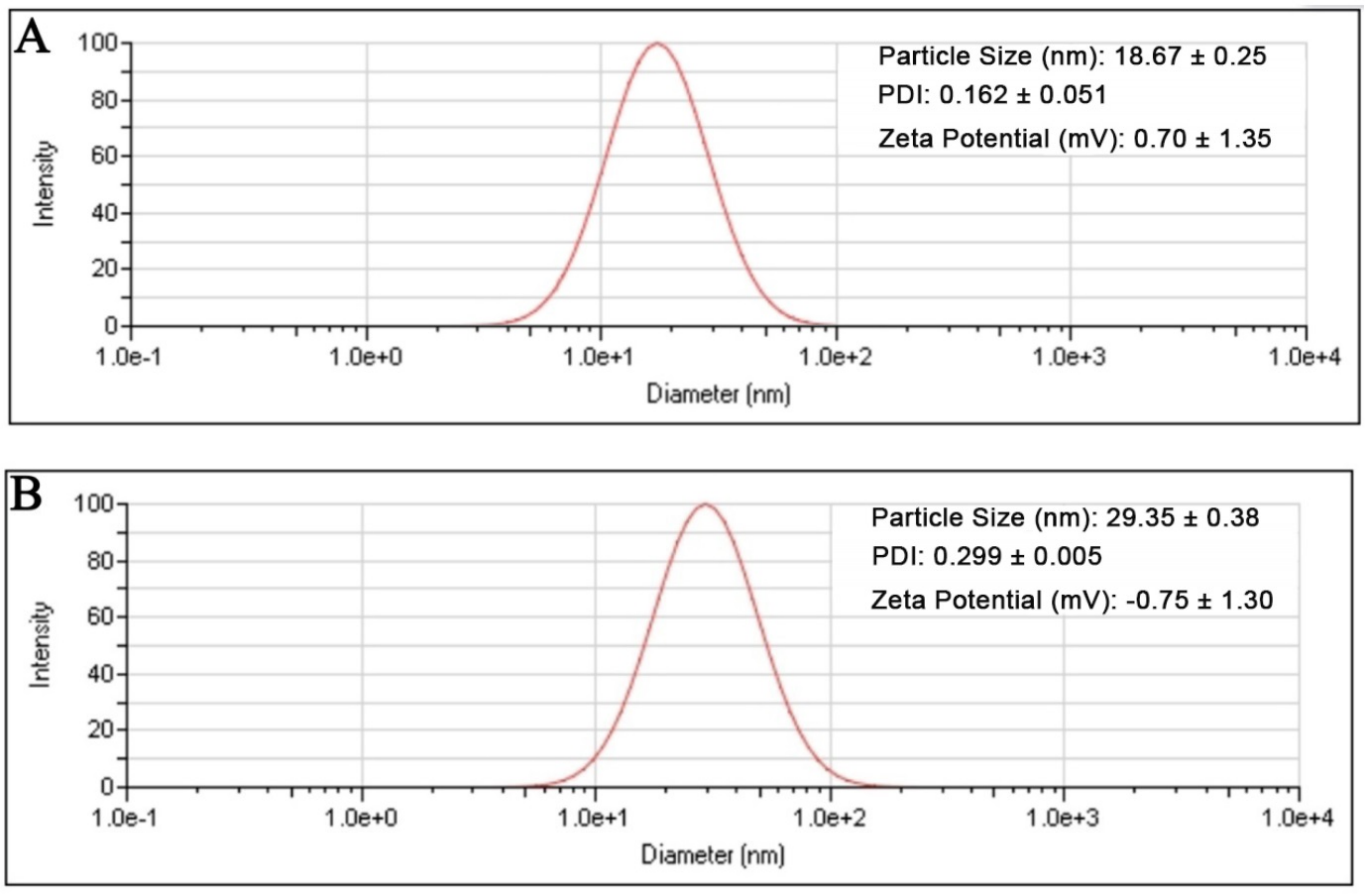
Particle size distributions of the blank polymeric micelles PMs** (A)** and UA-loaded polymeric micelles UA-PMs **(B)** determined by dynamic light scattering method.

**Figure 2:**
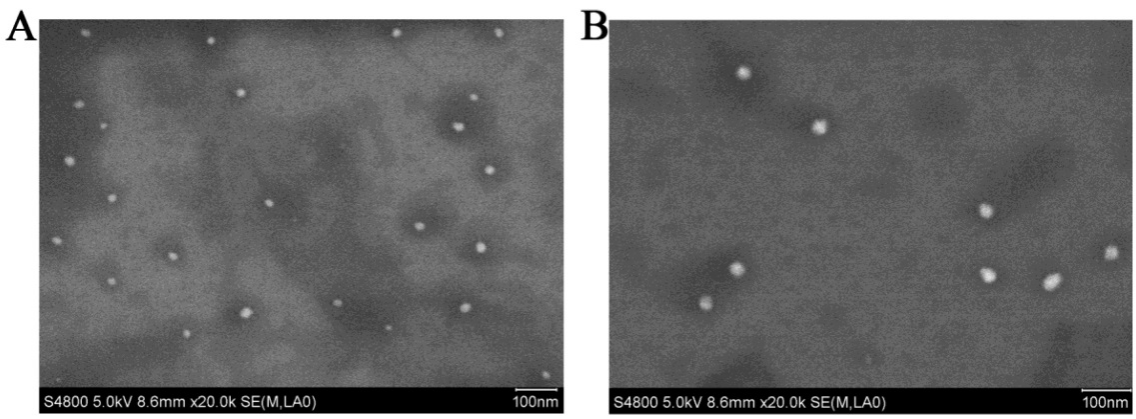
Scanning electron micrographs of blank PMs **(A)** and UA-PMs **(B)** viewed in a S-4800F (HITACHI) scanning electron microscope under the parameter setting of HV 5.0 kv, mag 20.0k ×, WD 8.6 mm and mode SE, respectively.

The result showed that the preformed blank PMs and UA-PMs prepared by thin film dispersion method were clear and transparent solution. If the micellar solution was stored at 4 °C condition, no visible precipitate appeared within 24 h. As shown in Figure 1A, the particle size of blank PMs and UA-PMs is in accordance with a normal distribution. The average particle size of blank PMs was 18.67 ± 0.25 nm with a PDI value of 0.162 ± 0.051 and the zeta potential was 0.70 ± 1.35 mV. For the UA-loaded polymer UA-PMs (Figure 1B), it increased to 29.35 ± 0.38 nm with a PDI value of 0.299 ± 0.005 and its zeta potential is -0.75 ± 1.30 mV. Significant difference in particle size was observed between blank PMs and UA-PMs (P < 0.0001). Moreover, all the PDI data were less than 0.3, which suggested the uniformity of all particles. The morphology detection indicated that most of blank PMs (Figure 2A) and UA-PMs (Figure 2B) are spherical. Moreover, the particle size of UA-PMs is larger than that of blank PMs. In summary, these results suggested that we have successfully prepared a relatively smooth spherical polymer micelle.

### *In vitro* release characteristic of UA-PMs

**Figure 3:**
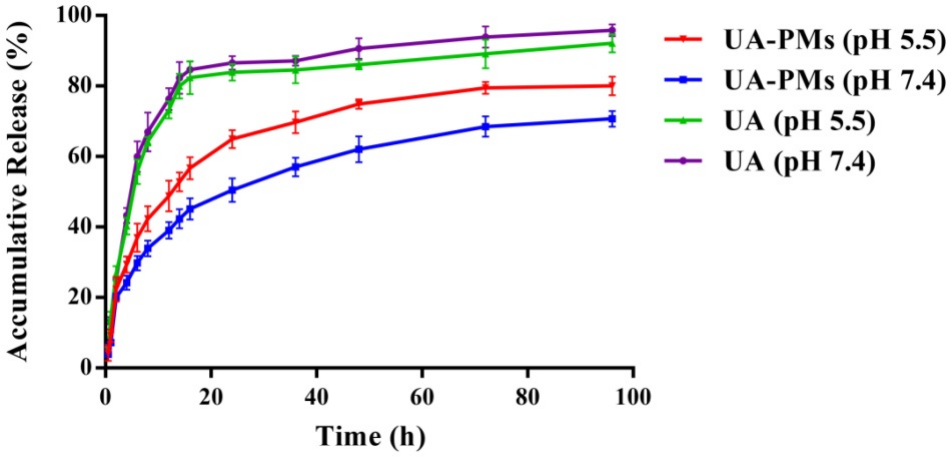
*In vitro* cumulative release profile of UA from UA-loaded polymeric micelles UA-PMs in PBS solution (pH 7.4 or pH 5.5) at 37 °C, compared with free UA. Results were presented as mean ± S.D. (n=3).

It is known that tumor is associated with the faintly acidic microenvironment 42-44. The *in vitro* release behavior of UA-PMs was evaluated at 37 °C using PBS as releasing medium at pH 7.4 and pH 5.5 conditions (Figure 3). Approximately 65 % of UA was released from UA-PMs within 24 h at pH = 5.5 condition. In contrast, only approximately 50% of UA was released within 96 h at pH 7.4, which demonstrated that UA PMs could increase the accumulation of UA in the tumor site. The major UA was released from UA solution within 24 h with an accumulative release rate of more than 80% at pH = 7.4 or pH = 5.5. However, about 65% of UA was released from UA-PMs at pH 5.5 and 50% of it was released at pH 7.4. No dramatic initial burst was observed over the same period, which indicated that the developed micelles exhibited significant sustained-release behaviors. This may be due to the reason that UA was embedded into the hydrophobic core and the released mechanism of UA from the micelle formulations might be concerned with the drug diffusion and the disintegration of polymer material. During the releasing process, the media gradually got into the micellar interior to make UA dissolve, and the carrier material be destroyed. Subsequently, UA was released from UA-PMs with a slow rate. This result is consistent with the previous report 42-44.

### Cell-proliferation and cell-migration inhibition of UA-PMs on HepG2 cells

**Figure 4:**
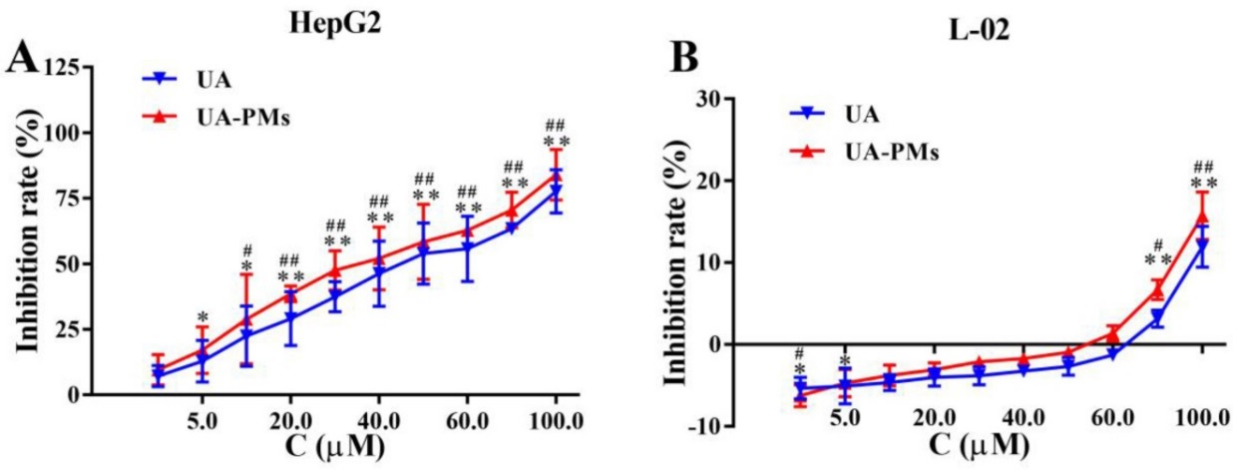
The *in vitro* cell-growth inhibition effect resulted from MTT assay on human hepatocellular carcinoma cell line HepG2 **(A)** and human normal liver cell line L-02 **(B)**, respectively. All cells were treated separately with various concentrations of UA and UA-PMs for 24 h. Each data point represents mean *±* standard deviation based on five repetitive measurements in three independent experiments. Results are presented as mean ± SD with *P< 0.05 or **P< 0.01 between the treated groups and saline group, ^#^*P*< 0.05 or ^##^*P*< 0.01 between the treated groups and the 5-FU group.

To investigate the therapeutic potential of UA-PMs, HepG2 and L-02 cells were incubated with different concentration of UA or UA-PMs for 24 h and the cell proliferation was measured by MTT assay. As shown in Figure 4A, the proliferation of HepG2 cells was significantly inhibited by UA and UA-PMs in dose-dependent manners, in which the IC50 values of UA and UA-PMs on HepG2 at 24 h were 43.2 ± 5.01 and 37.28 ± 2.44 μmol/L, respectively. This result indicated that UA-PMs could enhance the cell-growth inhibition effect against HepG2 cells compared to the free UA. However, in Figure 4B, treatments of both UA and UA-PMs did not obviously affect the cell proliferation with less than 20% of inhibition rate, implying that both of them had no cytotoxicity to the human normal liver cell line L-02. Meanwhile, both UA and UA-PMs showed bi-directional modulatory effect on the growth of L-02 cells, promoting the cell proliferation at low concentration but inhibiting it at high concentration.

**Figure 5:**
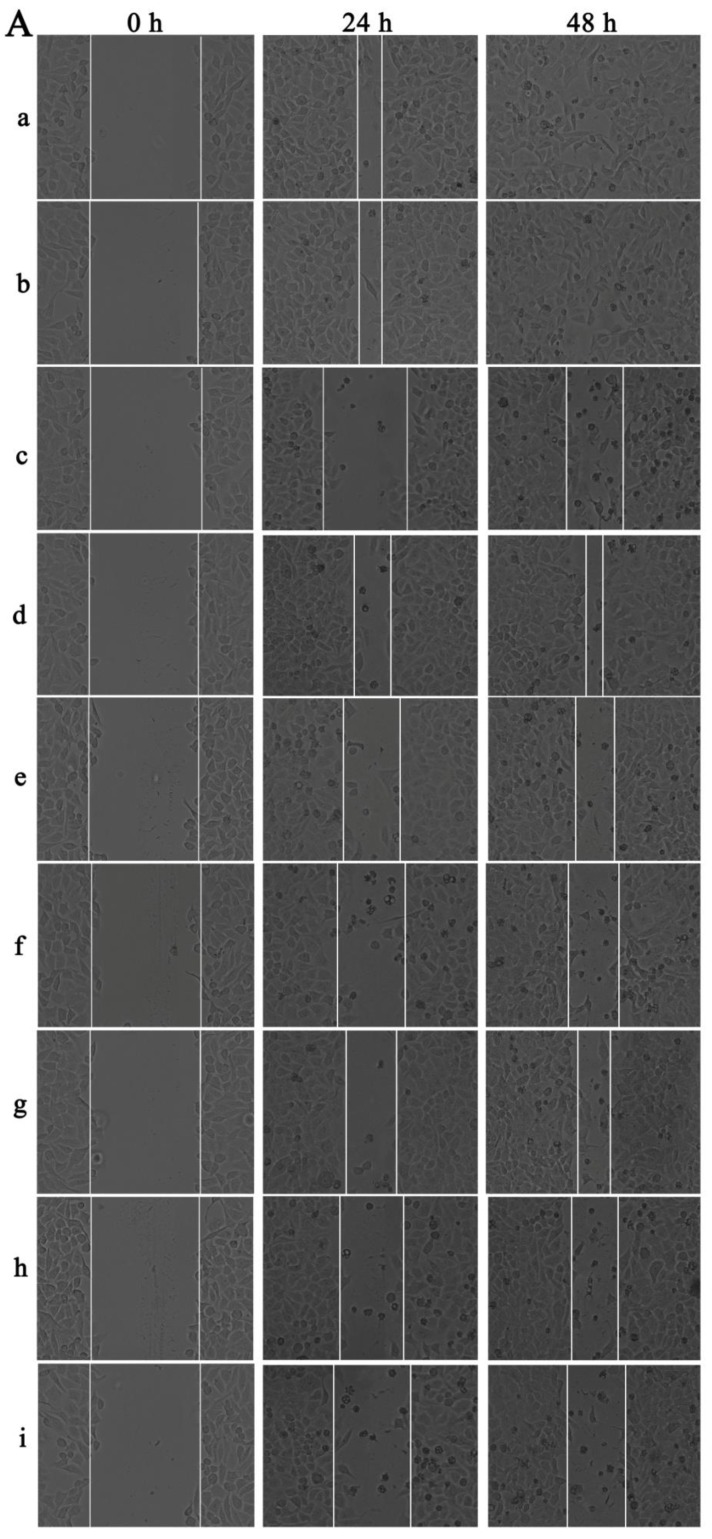

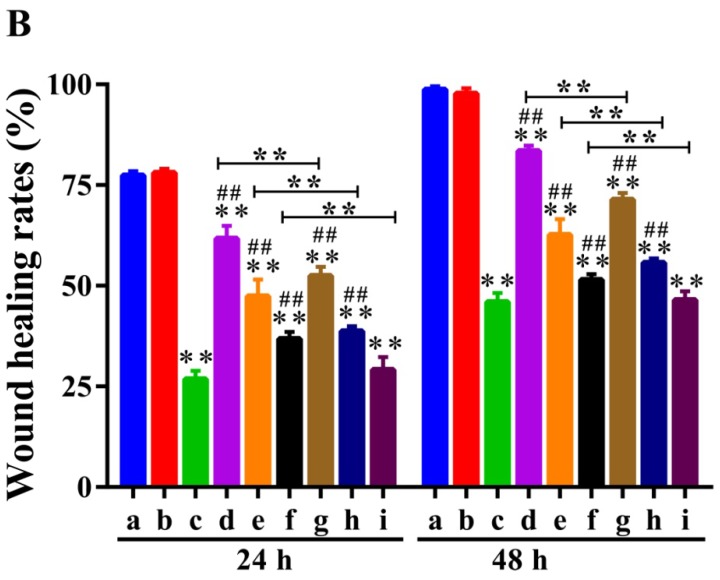
Qualitative (A) and quantitative (B) analysis of the wound healing assay on HepG2 cells. Cells were seeded in 6-well plates and incubated for overnight. A linear area of attached cells was removed by a pipette tip before treatment with different formulations as: **(a)** Saline, **(b)** Empty PMs, **(c)** 5-FU (20 μmol/L), **(d)** UA (20 μmol/L), **(e)** UA (40 μmol/L), **(f)** UA (80 μmol/L), **(g)** UA-PMs (20 μmol/L), **(h)** UA-PMs (40 μmol/L), **(i)** UA-PMs (80 μmol/L). HepG2 cells were photographed at different time point of 0 h, 24 h and 48 h (100 ×). Meanwhile, the scratch width (SW), distance between the cell fronts on either side of the wound, was measured. Each experiment was performed at least three times and results are presented as mean ± SD with*P< 0.05 or **P< 0.01 between the treated groups and saline group, ^#^*P*< 0.05 or ^##^*P*< 0.01 between the treated groups and the 5-FU group, ^*^P< 0.05 or ^**^P< 0.01 between UA and UA-PMs at the same concentration.

The wound-healing assay was performed to determine the inhibition effect of UA-PMs on the cell metastasis of HepG2. It can be seen from Figure 5A & 5B that after incubation for 48 h, scratches in both saline group and the blank PMs group were almost covered by cells and there was no obvious difference between these two groups on the relative wound area (*P* > 0.05). However, the scratches in the other treatment groups recovered more slowly the cell migrations were reduced compared with the saline negative control and the blank PMs group at both 24 h and 48 h after treatments (**P< 0.01). Compared with the 5-FU group, all treatment groups except the high-dose group of UA-PMs (^##^*P*< 0.01) showed significant differences, in which the high-dose UA-PMs (80 μmol/L) showed same inhibition effect as the positive control of 5-FU (*P* > 0.05) at both 24 h and 48 h. Furthermore, we found the wound healing rate in UA-PMs groups was lower than that in the UA groups at the same concentrations. These results revealed that the migratory potential of HepG2 cells treated with UA-PMs was significantly reduced in a concentration-dependent manner compared to UA groups (**^★★^***P*< 0.01).

### Antitumor efficacy of UA-PMs against H22 murine hepatoma

### Tumor growth inhibitory effect

Following the *in vitro* cell proliferation and cell migration studies about HepG2 cells and *in vivo* acute toxicity study of UA-PMs as shown in supplementary materials, pharmacodynamics experiments were carried out to evaluate the antitumor activities of UA-PMs *in vivo*. On day 5 after H22 tumor cells were implanted, different formulations taking saline as negative control and 5-FU 25 mg/kg as positive control were injected intraperitoneally into mice every other day for six times.

**Figure 6:**
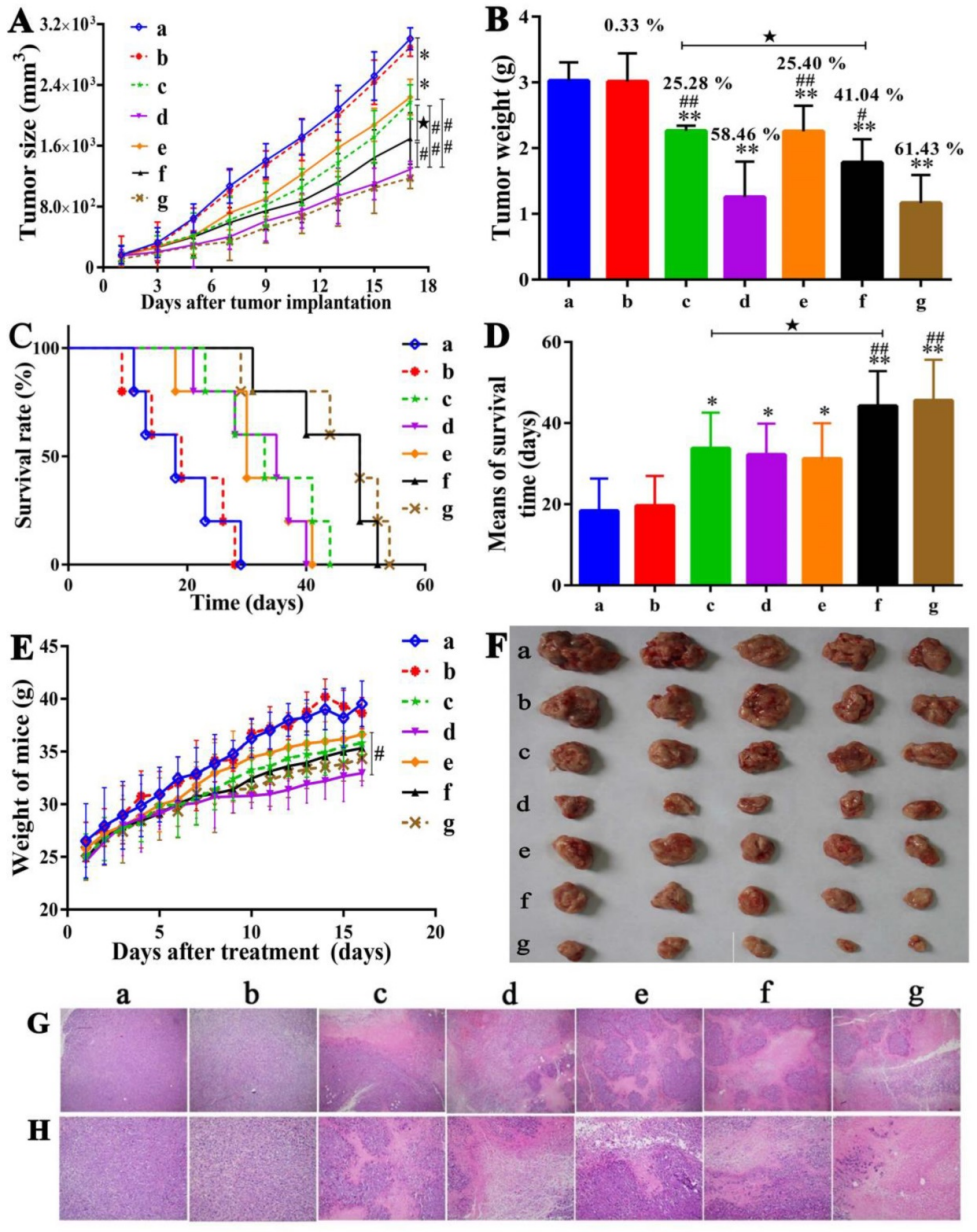
Antitumor effects of UA-PMs on H22 xenograft model. The H22 tumor-bearing mice in each group were treated every other day for 6 times from the 5th day on after inoculation with the different formulations as: **(a)** Saline, **(b)** Empty PMs, **(c)** UA (50 mg/kg), **(d)** 5-FU (25 mg/kg), **(e)** UA-PMs (25 mg/kg), **(f)** UA-PMs (50 mg/kg), **(g)** UA-PMs (100 mg/kg). Results described the mean tumor size of mice **(A)**, tumor weight **(B)**, survival curve of tumor-bearing mice **(C)**, mean for survival time **(D)** and weight of mice **(E)**. Data are shown as the mean ± SD in each group (n=5). Significant differences were observed between treated groups *and* saline (*P< 0.05, **P< 0.01), treated groups and 5-FU group (^#^*P*< 0.05, ^##^*P*< 0.01), UA and UA-PMs (**^★^***P* < 0.05) at the concentration of 50 mg/kg.

As shown in Figure 6A & 6B & 6F, tumor growths in the mice treated respectively with UA-PMs 100 mg/kg and 5-FU 25 mg/kg were inhibited significantly compared to the control animals that were treated with saline and blank PMs, respectively (p < 0.01). The mice treated with UA 50 mg/kg and UA-PMs 25 mg/kg appeared to exhibit moderate tumor growth inhibition. The formulation of UA-PMs inhibited the tumor growth in a dose-dependent manner.

Moreover, the tumor inhibition rate of 5-FU 25 mg/kg and UA-PMs 100 mg/kg is 58.46% and 61.43%, respectively, showing no significantly statistical difference between them (*P*> 0.05). Treatments of UA-PMs with different concentration of 100, 50 and 25 mg/kg led to a tumor inhibition rate of 61.43%, 41.04% and 25.40%, respectively, in which the obvious differences were shown between them with ^##^*P*< 0.01 and ^#^*P*< 0.05, respectively. Meanwhile, UA-PMs produced stronger tumor inhibition than free UA did at the same concentration of 50 mg/kg.

### Survival analysis

The survival curve was plotted by Graphpad prism software. As shown in Figure 6C & 6D, the actual survival time of mice in all treatment groups was longer than that of mice in the saline group and the blank PMs group. Moreover, the survival time of mice treated separately with UA-PMs 50 mg/kg and UA-PMs 100 mg/kg was increased to 44.2 ± 8.6 and 45.6 ± 10.0 days, respectively. Great differences were also observed in 5-FU 25 mg/kg vs UA-PMs 50 mg/kg, and 5-FU 25 mg/kg vs UA-PMs 100 mg/kg (^##^*P*< 0.01). The mice treated with UA-PMs 50 mg/kg showed a longer survival time than that treated with UA-PMs 25 mg/kg (**^★^***P*< 0.05). Moreover, at the same concentration of 50 mg/kg, UA-PMs obviously prolonged the mean survival times compared to free UA (**^★^***P*< 0.05). The weight of mice in each group reported everyday (Figure 6E) after the treatment increased gradually but the mice treated with 5-FU showed the lowest increase of weight. It may be due to the toxicity as proved in the supplementary materials.

### Pathological analysis of tumor tissues by H&E staining

In order to further validate the antitumor activity of UA-PMs, pathological analysis of tumor tissues was performed by H&E assay. As shown in Figure 6G (100 ×) and 6H (200 ×), the tumor necrosis was not obvious in the saline group and the blank PMs group but there were typical necrosis, such as nuclear fragmentation, contraction, and dissolution in the tumor issue of mice treated with 5-FU, UA and UA-PMs.

Compared with saline control, the average necrosis rate of cell populations was about 31.25% for UA 50 mg/kg, 84.75% for 5-FU, 29.17% for UA-PMs 25 mg/kg, 56.25% for UA-PMs 50 mg/kg, 77.08% for UA-PMs 100 mg/kg. Moreover, most of the cancer cells in high-dose of UA-PMs group (100 mg/kg) showed high degree of differentiation, which may be the evidence for the higher antitumor activity of UA-PMs. This result is consistent with that of antitumor effect mentioned above, suggesting that UA-PMs could cause cell necrosis of H22 and lead to the tumor growth inhibition.

## Discussion

In present study, we successfully developed the novel drug delivery system of UA-loaded polymeric micelles (UA-PMs) and revealed that UA-PMs significantly enhanced cell-growth and cell-migration inhibition effects against the human hepatocellular carcinoma cells HepG2 while showing no toxicity to both human normal liver cell line L-02 *in vitro* and the normal Kunming mice *in vivo*. Further, we found UA-PMs could suppress the tumor growth of H22 xenograft and prolong the survival time of the tumor-bearing mice. These results are consistent with the previous report 6, 8, 12, 13.

It is known that both critical micelle concentration (CMC) value and zeta potential are regarded as the important parameters for the stability of nanoparticle drug delivery system. If the absolute values of zeta potential of nanoparticle were more than 30 mV, it would be better in terms of dynamic stability. In this study, as shown in supplementary Figure S1, the CMC values of the blank PMs and UA-PMs were respectively 5.1 × 10^-3^, 2.3 × 10^-3^ mg/mL indicating that the micelles showed good stability. However, the zeta potential of micelles was about 0 mV. This may be due to the presence of a great quantity of PEG blocking on the surface of UA-PMs particle, which could decrease the surface tension between particles and further prevent vesicle aggregation, thereby resulting in a near-neutral zeta potential of the micelles and keeping the stability of the formulation 21.

In our acute toxicity study, we found the spleen coefficients (Supplementary table S1) of mice treated with high dosage of UA and UA-PMs were obvious higher than that of the control group but the histopathological result (Supplementary Figure S2) showed that no damage was observed in the spleens of mice. In one hand, the organ coefficient was calculated according to the ratio of organ weight and body weight. After the separate treatments with different formulations, body weight of the treated mice gained slowly and even showed a decreased trend, which may lead to the increase of spleen coefficients. On the other hand, the organ coefficient of immune organs could be used as a preliminary indicator for judgment of immune function. In consideration of the spleen as an important immune organ, the spleen coefficient can be used to evaluate the body immunity. Therefore, an increased spleen index may represent an increasing immune function.

It was reported that drug efficacy was seriously limited by poor solubility. The solubility problems are frequently encountered in the preparations of pharmaceutical dosage forms 38. UA is poor in water solubility, which limited its potential application in clinical therapy. Both considerations about pharmacokinetic and pharmacodynamic are equally important in increasing the biological effects and bioavailability of UA. Some researchers have been performed to improve the water-solubility of UA by chemical modifying, such as the non-covalent complex with hydrophilic cyclodextrins, the use of nanosuspensions or the preparation of surfactant solutions and PEGs 38, 45, 46. But among these researches, only moderate solubilizing capacity for UA has been obtained with the methods reported. In this study, we first took polymeric micelles as the carrier to load UA and successfully prepared PMs-UA. Meanwhile, in recent years, block copolymers have been developing very rapidly in the drug delivery formulations because of their versatile and flexible structure 16. In this novel drug delivery system of PMs-UA, mPEG-PLA is a kind of amphiphilic block copolymers with low immunogenicity, biodegradability and biocompatibility, in which PEG is the hydrophilic tail and PLA is the hydrophobic tail. This kind of amphiphilic block copolymer tends to self-assemble automatically and forms the unique core-shell structure consisting of a hydrophobic inner core and a hydrophilic corona outside. Therefore, UA was gulfed into the hydrophobic inner core and was protected from degrading, which further led to the controlled release of UA from PMs-UA.

To investigate whether the preformed UA-PMs could be used in anti-hepatoma therapy, the *in vitro* anticancer activity of UA-PMs was tested against HepG2 cell line. The *in vitro* cell growth inhibition study indicated that UA-PMs showed the obvious inhibition effect on the HepG2 cells. And the IC50 value of UA-PMs was lower than that of the native UA at 24 h or 48 h showing the higher inhibitory effect compared with free UA at the same dosage. At the same time, the scratch-healing experiment was conducted to investigate the inhibition of UA-PMs on HepG2 cells migration and the results indicated that UA-PMs showed stronger inhibitory effect on the cell migration than UA with the same concentration. These results are consistent with the previous report 14. Further, the anti-tumor test* in vivo* was taken to evaluate the anti-hepatocarcinoma effect of UA-PMs. In this experiment, UA-PMs at dose of 25, 50, 100 mg/kg were treated by i.p. administration against H22xenograft mice every other day for 17 days. From the results, UA-PMs were more effective for inhibiting tumor growth and prolonging survival times, compared with saline and free UA groups. Although 5-FU showed a similar inhibitory effect on HCC as UA-PMs at the same concentrations, the survival time of mice was bad in the treatment of 5-FU. It might because 5-FU had a large side effect on the heart of the treated mice.

These results from the cytotoxicity study *in vitro*, acute toxic study and antitumor activity *in vivo all* proved our hypothesis that UA-PMs could overcome the disadvantages of UA and get a sustained release of UA from UA-PMs thus enhancing antitumor effect on hepatocellular carcinoma. However, for the further clinical application, the formulation should be further optimized to improve the entrapment efficiency, drug-loading rate. On the other hand, maybe there is another way to develop UA formulation to conjugate UA with the amphiphilic block copolymers to form UA-PEG-PLA and further use it to prepare micelles 6, 12-15.

## Supplementary Material

Supplementary figures and tables.
