# Supplementary material for: Polymeric micelles loading with ursolic acid enhancing anti-tumor effect on hepatocellular carcinoma
Source: J Cancer. 2019 Oct 6;10(23):5820–31. doi: 10.7150/jca.30865 (PMC6843872; doi:10.7150/jca.30865)
Supplement: Supplementary file 1 — Supplementary figures and tables. [file jcav10p5820s1.pdf]

Supplementary material

Materials and methods

Stability study of UA-PMs

To estimate the physical stability of UA-PMs, the freshly prepared UA-PMs (n = 3) were placed into the glass vials and stored at refrigerator at 4 °C for 30, 60 and 90 days, respectively. Meanwhile, UA-PMs were also stored at the condition of 25 °C ± 2 °C / 60% RH ± 5% RH f for the acceleration test. The drug-leakage rate was monitored using the HPLC method described above. Meanwhile, characterization of morphological changes of UA-PMs *in vitro* was observed under optical microscope once every hour within 12 h from the beginning of the storage.

In addition, the stability assay in cell culture media was performed as following: three batches of newly prepared UA-PMs (2 mL) were respectively mixed with the same volume of cell culture media and placed into dialysis bags (MWCO = 2000) which were suspended in 100 mL PBS solution (pH = 7.4). The sealed vials were placed in a water bath (37 °C) with a stirring speed of 50 rpm. At the various time intervals of 0, 0.5, 1, 2, 6, 12, 14 and 24 h, 1 mL of the solution was withdrawn and replaced with fresh PBS and repeated for 3 times, with PBS as controls. The samples were measured by HPLC method as described above to

calculate the accumulative percentage of leakage.

Critical micelle concentration determination of UA-PMs

The critical micelle concentration (CMC) of PMs and UA-PMs in water were determined by fluorescence technique using pyrene as a fluorescence probe. Specifically, 10 mL of micellar solutions with a serial concentration of 1.0×10^{-5}, 2.0×10^{-5}, 5.0×10^{-5}, 1.0×10^{-4}, 2×10^{-4}, 5×10^{-4}, 1.0×10^{-3}, 2.0×10^{-3}, 5.0×10^{-3}, 1.0×10^{-2}, 2.0×10^{-2}, 5.0×10^{-2}, 1.0×10^{-1}, 2×10^{-1}, 5.0×10^{-1}mg/mL were added separately into volumetric flasks containing pyrene (final concentration 6×10^{-6}mol/L). The samples were sonicated for 30 min and incubated for overnight at room temperature in dark room to equilibrate the pyrene partition between the water and micelles. For fluorescence measurement, the emission spectra of pyrene were recorded from 300 to 500 nm with a scanning rate of 1200 nm/min. and the excitation wavelength was 335 nm at room temperature. Meanwhile, the excitation and emission bandwidth were 5 and 2.5 nm, respectively. Fluorescence intensity at emission wavelength of 373 nm (I_{373}) and 393 nm (I_{393}) were measured by F-2500 fluorescence spectrophotometer at room temperature. The intensity ratio ($I_{373\ nm}/I_{393\ nm}$) of pyrene fluorescence bands was plotted against the micelle concentration and CMC value was obtained from the intersection of the tangent to the curve at the inflection with the horizontal tangent.

Acute toxicity assessment of UA-PMs in mice

The acute toxicity of UA-PMs *in vivo* was evaluated on KM mice, and the animals were randomly divided into eleven groups (n=10). The negative group received only the vehicle (sterile normal saline) by the intraperitoneal injection. The other nine groups were treated intraperitoneally with blank PMs (500 mg/kg) and UA, UA-PMs at various doses (125, 250, 500, 750 mg/kg) at one time with 5-FU as the positive control. All groups were administered in a volume of 0.5 mL. In the following week, mice were keenly observed for the adverse effects. The weight of the mice was also measured regularly as an indicator of acute toxicity. Blood samples were collected from the orbital venous plexus of mice on day -1 (before treatment), and on day 3, day 7 post-administration for liver and kidney function detection of mice. Detection of serum alanine aminotransferase (ALT), aspartate aminotransferase (AST), blood urea nitrogen (BUN) and creatinine (Cr) were conducted with the automated biochemical analyzer (LabMax 240, Labtest, Brazil) in the Affiliated Hospital of Southwest Medical University (Luzhou, China). The mice were sacrificed on day 7 after treatment. The heart, liver, lung and kidney of mice were rapidly excised, washed with saline, weighed and fixed with 10 % formalin and dehydrated with a graded series of ethanol and xylene, then embedded in paraffin. Slices (5-6 μm) of tissues were prepared from paraffin blocks, and stained with hematoxylin and eosin (H&E) after dewaxed and rehydrated. Histopathological

changes were observed and photographed by microscopy. The organ coefficient was estimated according to the following formula as: organ coefficient (%) = organ weight / body weight × 100%.

Results

Stability analysis

We used the optimized chromatographic conditions to quantitatively determine UA. The solution kept clear and showed no changes between 0 to 12 h when UA-PMs stored in 4 °C. The physical stability study indicates that UA-PMs are stable with an average drug-leakage rate of 2.5% during the storage within 30 days. However, the average drug-leakage rate is increased to 18% at the day 60 of storage and obvious precipitates were formed at the end of 90-day storage with an average drug-leakage rate of 48%. Meanwhile, we also found in the acceleration test, when UA-PMs were stored at the condition of 25°C ± 2°C/60% RH ± 5% RH, the average drug-leakage rate is 1.5% after 5-day storage, 10% after 15-day storage. After a 30-day storage, the solution became un-clear and the obvious precipitates were formed. Therefore, UA-PMs should be prepared with freezing dry technology for long-term storage.

For media stability test, there is no bursting release and the accumulative percentage of leakage from UA-PMs at the different time points of 0.5, 1, 2, 6, 12, 14 and 24 h are 0.00 ±

0.03 %, 0.00 ± 0.59 %, 19.15 ± 1.21 %, 30.06 ± 1.29 %, 41.21 ± 3.32 %, 46.13 ± 3.31 % and 53.22 ± 2.15 %, respectively. This result is consistent with that from the in vitro release study, in which UA-PMs was not mixed with culture media containing 10% fetal bovine serum. That means UA-PMs shows the same drug-release profile with or without serum presence, which indicated that UA-PMs could keep stable in cell culture media, the blood-mimicking conditions.

CMC result of ***UA-PMs***

To evaluate the stability of UA-PMs and PMs, the CMC values of them were determined through using pyrene as the fluorescent probe. As a result shown in **Supplement Figure 1A & B**, the CMC values of the blank PMs and UA-loaded PMs were 5.2×10^{-3} and 2.4×10^{-3} mg/mL, respectively. Both of them were small, which indicated that the preformed polymer micelles could be stable.

Acute toxicity result of UA-PMs

Viscera index detection

The *in vivo* acute toxicity of UA-PMs was evaluated after a single intraperitoneal administration of the different drugs in the normal KM mice. Throughout the test period, compared to the positive control group (5-FU, 250 mg/kg), the other ten groups did not

produce any noticeable side effects on the activities of all tested mice. The food and water consumption remained as normal. As shown in **Table S1**, compared with the saline group (*P*> 0.05), after treatments with the blank PMs, UA and UA-PMs, the viscera index of heart, lung and kidney exhibited no significant difference. Compared to the saline group with a live index of 5.623 ± 0.206%, the high-dose UA group with 750 mg/kg increased it to 6.258 ± 0.259% ($^{**}P < 0.01$); but the other indices increased slightly and there is no significant difference ($P > 0.05$). For the spleen coefficient, it significantly increased in the treatments of UA 500 mg/kg, UA 750 mg/kg and UA-PMs 750 mg/kg, with a statistically significant difference compared with the saline group ($^{*}P < 0.05$, $^{**}P < 0.01$). These results indicated that both UA and UA-PMs had no obvious side effects on heart, liver, lung, and kidney within a certain concentration range. However, in the treatment of 5-FU 250 mg/kg, the organ coefficient of heart obviously increased ($^{*}P < 0.05$) compared with the saline group, which showed serious heart-toxicity to the tested mice.

Serum biochemical parameters

To further evaluate the potential toxicity of UA-PMs to the liver and kidney of mice, serum biochemical indices were analyzed as shown in **Table S2** For the liver function markers including AST and ALT, compared with saline group only in the treatment group of UA 750 mg/kg, both ALT and AST decreased significantly on day 7 with $^{**}P < 0.01$ and $^{*}P < 0.05$,

respectively. In the other treated groups, both AST and ALT keep stable and no significant differences were observed ($P > 0.05$). On concerning of the kidney function indicators of BUN and Cr, no significant differences were observed between the drug experimental groups and the saline control group on both day 3 and day 7 after treatment ($P > 0.05$). These results suggested that UA-PMs had no kidney toxicity to the tested mice and only the high dose of it showed side effect to the live of the tested mice.

Histopathological analysis

The histopathological study on heart, liver, spleen, lung and kidney was conducted to observe whether there are pathological changes for estimating the potential toxicity of UA-PMs. As described in **Figure S2**, significant muscular rupture was found in the heart section of mice treated with 5-FU but no significant lesion in cardiac structure was observed in the other treatment groups. For the liver section, the pathological changes were shown in the high-dose UA treatment group, appearing the portal area inflammation and necrosis, inflammatory cell infiltration and congestion. No significant pathological changes of liver were observed in the other treatment groups. Regarding of spleen, lung, kidney sections, all the groups showed no obvious lesions.

For both lung and kidney, the histopathological result was consistent with the detection about viscera index, which indicated that UA-PMs showed no toxic effects in these two

organs. Comparing the results about spleen toxicity to mice after treatment with UA 500 mg/kg, UA 750 mg/kg and UA-PMs 750 mg/kg, we found the result from viscera index was not consistent with that from histopathological analysis, in which the spleen index was obvious increased (**Supplement table S1**) but the histopathological section was normal (**Supplement Figure S2**). This phenomenon may because the spleen index is not sure to be relative with spleen injury but it has relationship with the immune function as mentioned in discussion.

From these results, we can see that UA-PMs at a dose of 125, 250 and 500 mg/kg showed no toxic effect on normal mice and these doses may be the better choices in our further experiments, but it still needs to be confirmed via the following study about antitumor activities.

Supplement table 1

Table S1 Organ coefficient of mice treated with different formulations.

Groups	Heart (%)	Liver (%)	Spleen (%)	Lung (%)	Kidney (%)
Saline	0.535 ± 0.083	5.623 ± 0.206	0.262 ± 0.045	0.637 ± 0.157	1.367 ± 0.050
Blank PMs	0.596 ± 0.037	5.785 ± 0.717	0.291 ± 0.016	0.707 ± 0.124	1.388 ± 0.179
5-FU (250 mg/kg)	0.671 ± 0.046*	5.923 ± 0.759	0.320 ± 0.131	0.766 ± 0.102	1.293 ± 0.125
UA (125 mg/kg)	0.558 ± 0.002	5.451 ± 0.456	0.292 ± 0.056	0.779 ± 0.064	1.316 ± 0.098
UA (250 mg/kg)	0.593 ± 0.039	5.777 ± 0.576	0.341 ± 0.065	0.701 ± 0.084	1.352 ± 0.170

UA (500 mg/kg)	0.578 ± 0.079	6.061 ± 0.790	0.394 ± 0.096*	0.816 ± 0.137	1.378 ± 0.118
UA (750 mg/kg)	0.582 ± 0.067	6.258 ± 0.259**	0.454± 0.058**	0.775 ± 0.293	1.414 ± 0.255
UA-PMs (125 mg/kg)	0.527 ± 0.069	5.406 ± 0.387	0.280 ± 0.063	0.792 ± 0.093	1.341 ± 0.008
UA-PMs (250 mg/kg)	0.502 ± 0.104	5.379 ± 0.273	0.341 ± 0.094	0.678 ± 0.090	1.298 ± 0.137
UA-PMs (500 mg/kg)	0.528 ± 0.036	5.835 ± 1.229	0.354 ± 0.144	0.690 ± 0.054	1.310 ± 0.139
UA-PMs (750 mg/kg)	0.575 ± 0.085	6.053 ± 0.688	0.541 ± 0.208*	0.794 ± 0.104	1.353 ± 0.015

Results were presented as mean ± S.D. (n=5). The organ coefficient was estimated according to the following formula as: organ coefficient (%) = organ weight / body weight × 100. Symbols represented statistical significance compared with saline control group (*$P < 0.05$, **$p < 0.01$).

Supplement table 2

Table S2 The blood biochemical parameters of serum from mice treated with different formulations

Parameters	Formulations	Day -1	Day 3	Day 7
ALT (U/L)	Saline	39.40 ± 1.13	45.20 ± 7.77	44.73 ± 8.82
	Blank PMs	40.50± 0.42	46.50 ± 7.38	47.00 ± 8.03
	5-FU (250 mg/kg)	35.30 ± 4.67	47.93 ± 3.30	49.33 ± 3.59
	UA (125 mg/kg)	31.80 ± 9.62	44.96 ± 3.23	44.13 ± 8.25
	UA (250 mg/kg)	39.45 ± 0.49	42.20 ± 8.95	39.17 ± 3.71
	UA (500 mg/kg)	34.05 ± 8.56	40.97 ± 6.85	34.63 ± 5.89
	UA (750 mg/kg)	37.25 ± 2.07	36.90 ± 3.84	27.78 ± 4.00**
	UA-PMs (125 mg/kg)	33.35 ± 8.13	43.27 ± 1.65	40.63 ± 5.93
	UA-PMs (250 mg/kg)	42.85 ± 6.80	38.2 ± 4.70	37.25 ± 3.83

	UA-PMs (500 mg/kg)	39.90 ± 5.80	38.23 ± 2.52	36.57 ± 4.26
	UA-PMs (750 mg/kg)	35.48 ± 4.59	36.40 ± 3.94	34.97 ± 4.29
AST (U/L)	Saline	154.70 ± 31.24	159.93 ± 19.82	153.87 ± 15.60
	Blank PMs	148.45 ± 37.97	154.35 ± 19.59	148.00 ± 23.48
	5-FU (250 mg/kg)	176.70 ± 18.04	144.10 ± 15.37	152.35 ± 29.14
	UA (125 mg/kg)	144.20 ± 19.66	154.93 ± 27.35	146.23 ± 31.06
	UA (250 mg/kg)	179.75 ± 21.71	159.13 ± 16.67	137.57 ± 21.61
	UA (500 mg/kg)	159.65 ± 14.30	151.83 ± 29.72	138.23 ± 27.68
	UA (750 mg/kg)	148.20 ± 10.61	143.03 ± 13.51	125.73 ± 16.70*
	UA-PMs (125 mg/kg)	154.75 ± 24.80	156.93 ± 11.66	150.50 ± 21.53
	UA-PMs (250 mg/kg)	156.85 ± 12.52	147.67 ± 26.04	143.37 ± 19.85
	UA-PMs (500 mg/kg)	150.45 ± 6.43	144.60 ± 17.27	133.77 ± 22.82
	UA-PMs (750 mg/kg)	147.40 ± 14.99	141.60 ± 19.75	137.30 ± 11.68
BUN	Saline	8.31 ± 0.64	8.58 ± 0.90	8.60 ± 1.44
(mmol/L)	Blank PMs	9.11 ± 0.82	9.28 ± 0.23	9.23 ± 1.53
	5-FU (250 mg/kg)	7.59 ± 0.83	8.44 ± 0.83	9.64 ± 0.94
	UA (125 mg/kg)	8.24 ± 1.68	9.29 ± 0.84	9.44 ± 1.29
	UA (250 mg/kg)	8.99 ± 0.37	9.90 ± 1.11	8.63 ± 1.37
	UA (500 mg/kg)	8.33 ± 0.33	8.09 ± 0.83	7.50 ± 1.25
	UA (750 mg/kg)	7.77 ± 1.06	10.12 ± 1.63	9.21 ± 0.67
	UA-PMs (125 mg/kg)	9.31 ± 1.52	9.61 ± 1.23	7.37 ± 1.23
	UA-PMs (250 mg/kg)	8.80 ± 0.25	9.67 ± 1.54	9.42 ± 0.35
	UA-PMs (500 mg/kg)	9.47 ± 0.93	7.78 ± 2.02	9.13 ± 1.48
	UA-PMs (750 mg/kg)	7.46 ± 1.27	8.32 ± 0.67	7.62 ± 0.76
Cr	Saline	38.70 ± 1.26	42.66 ± 2.20	39.08 ± 4.40
(μmol/L)	Blank PMs	36.60 ± 3.39	40.90 ± 3.48	42.68 ± 7.44
	5-FU (250 mg/kg)	37.32 ± 2.52	39.76 ± 2.30	34.80 ± 3.28
	UA (125 mg/kg)	36.97 ± 1.46	40.40 ± 3.52	38.68 ± 8.20

UA (250 mg/kg)	40.50 ± 1.26	41.46 ± 3.94	38.12 ± 8.24
UA (500 mg/kg)	39.00 ± 2.66	38.94 ± 2.98	37.20 ± 5.44
UA (750 mg/kg)	40.70 ± 1.71	42.40 ± 3.86	37.32 ± 1.24
UA-PMs (125 mg/kg)	37.68 ± 1.01	42.94 ± 2.48	38.40 ±3.48
UA-PMs (250 mg/kg)	37.56 ± 4.22	44.54 ± 2.90	37.20 ± 2.08
UA-PMs (500 mg/kg)	37.29 ± 1.39	39.84 ± 2.24	32.27 ± 5.28
UA-PMs (750 mg/kg)	43.05 ± 4.44	43.56 ± 2.63	34.12 ± 7.08

Results were presented as mean ± S.D. (n= 5). Symbols represented statistical significance compared with saline group (*$P < 0.05$). Abbreviations: ALT, alanine aminotransferase; AST, aspartate aminotransferase; BUN, blood urea nitrogen; Cr, creatinine.

Supplement figure 1

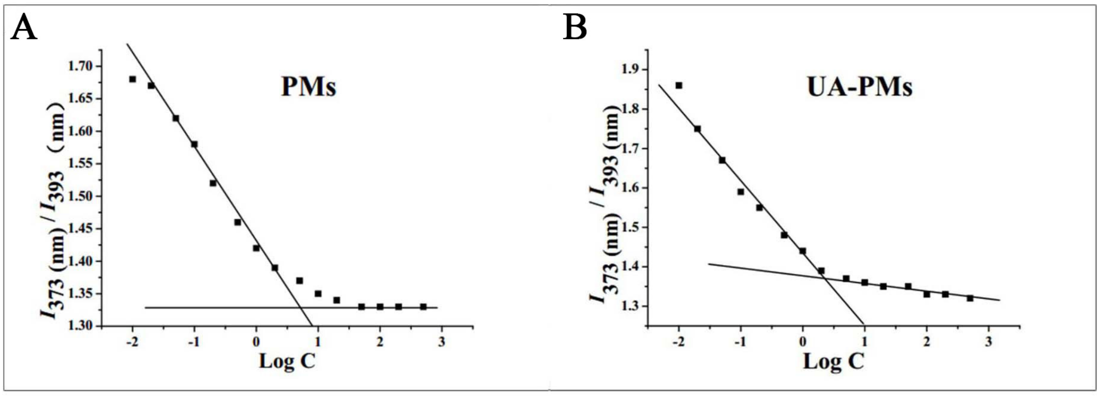

Figure S1. Critical micelle concentration (CMC) determination of blank polymeric micelles PMs (A) and UA-loaded polymeric micelles UA-PMs (B). CMC value was determined according to the concentration at the crossover point in the plots of the fluorescence intensity ratio (I_{373}/I_{393}) against the logarithm of micelle concentration (μg/mL).

Supplement figure 2

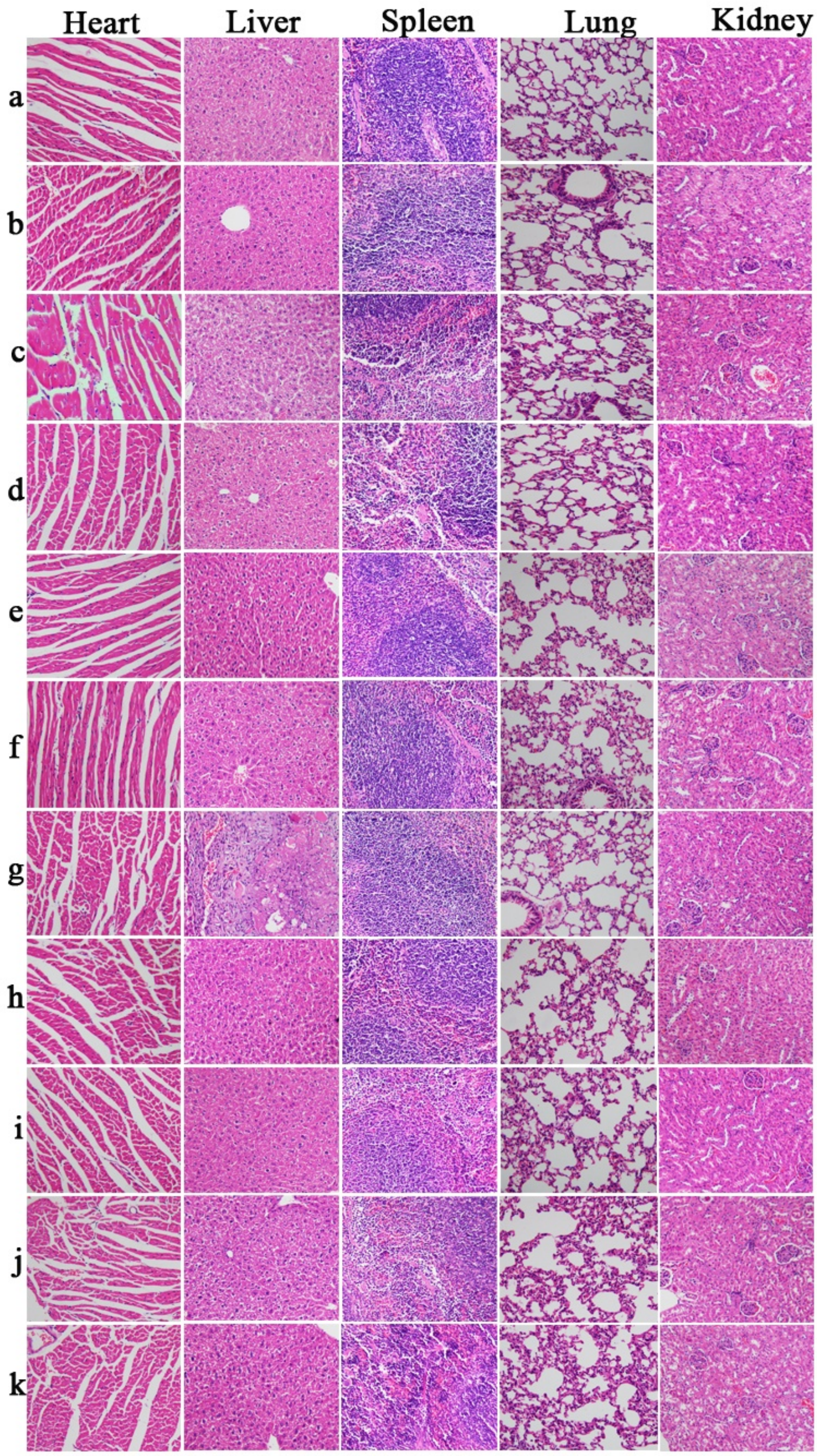

Figure S2. H&E results of histopathological sections of the heart, liver, spleen, lung and kidney from the KM mice treated by a single intraperitoneal injection of different formulations as: (a) Saline, (b) Blank PMs, (c) 5-FU (250 mg/kg), (d) UA (125 mg/kg), (e) UA (250 mg/kg), (f) UA (500 mg/kg), (g) UA (750 mg/kg), (h) UA-PMs (125 mg/kg), (i) UA-PMs (250 mg/kg) (j) UA-PMs (500 mg/kg), (k) UA-PMs (750 mg/kg).
